# Chronic Schistosomiasis Presenting with Migrating Pulmonary Manifestation after Recent COVID-19 Infection: HRCT Findings

**DOI:** 10.5334/jbsr.2758

**Published:** 2022-04-27

**Authors:** Stijn Laveaux, Stefaan Vandecasteele, Kris Van De Moortele

**Affiliations:** 1 UZ Leuven, BE; 2 AZ Sint Jan Brugge, BE

**Keywords:** Pulmonary schistosomiasis, HRCT, CT, Lung disease, parasitic

## Abstract

**Teaching Point:** Chronic pulmonary schistosomiasis should be suspected in symptomatic patients with an endemic background presenting with migrating pulmonary lesions on high resolution computed tomography scan.

## Case History

A 38-year-old woman with a Sierra Leonean background presented to our institution complaining of pleural pain, fever, and cough. She had a history of human immunodeficiency virus successfully treated with triple therapy and a recent subclinical COVID-19 infection. The patient had not been to Sierra-Leone for 10 years and there was no history of recent travelling or contact with sick patients or Tuberculosis patients.

Physical examination was unremarkable. Laboratory tests showed leukocytosis with eosinophilia (0.8 10.E9/l) and high CRP (33.7). Further investigation done with a urinary sample, broncho-alveolar lavage, and serological tests (syphilis, hepatitis, Q-fever) concluded negative.

**Figure 1:**
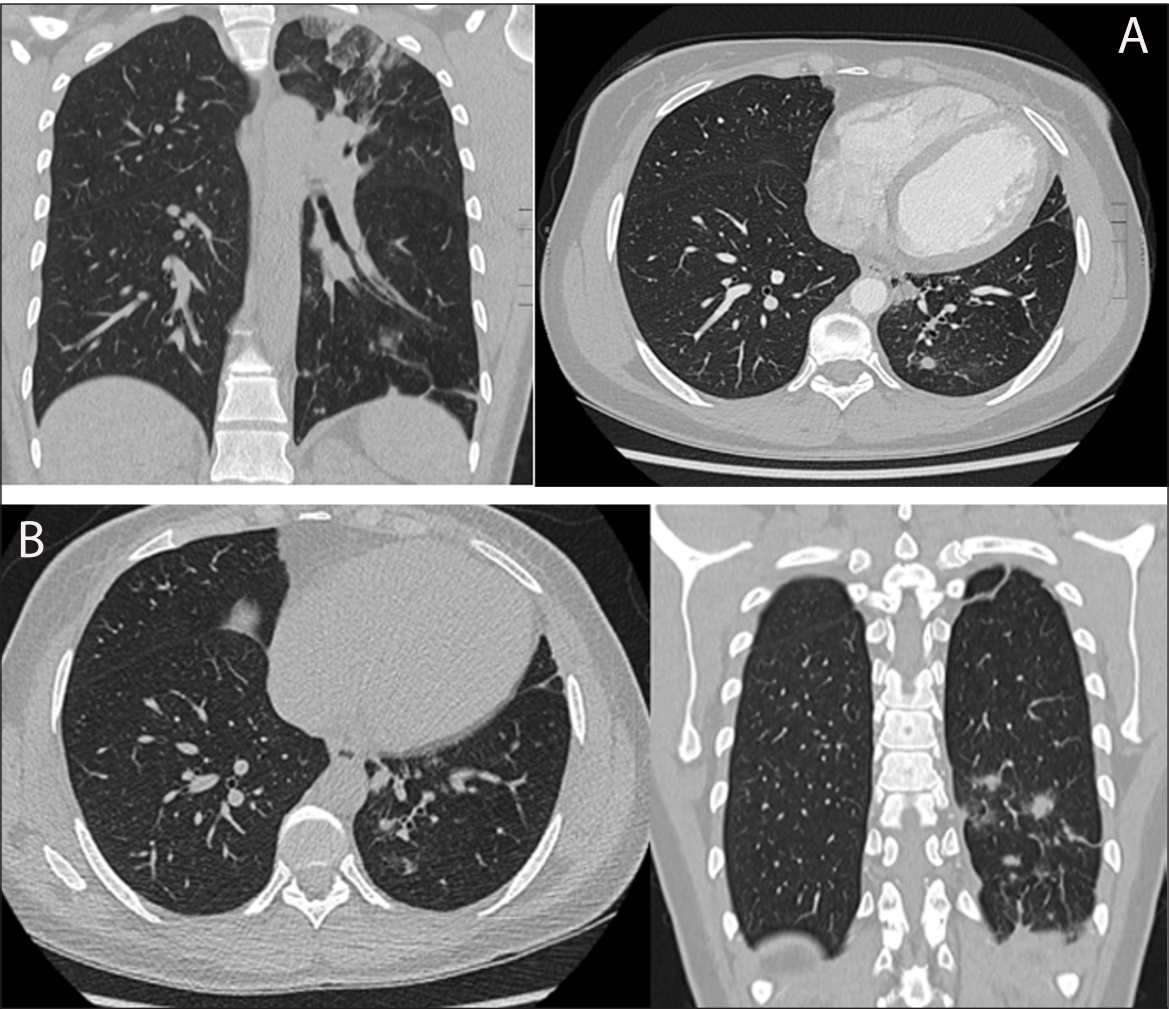


High resolution CT (HRCT) a few days after admission demonstrated multiple nodular zones of consolidation with surrounding ground glass in the left lower lobe (***Figure 1A***). No TBC-associated lesions or calcified nodes were observed. The first follow-up HRCT was scanned after seven days (***Figure 1B***) of watchful waiting without any medical therapy. These follow-up images demonstrated a spontaneous regression of some lesions where other nodular lesions demonstrated expansion compared to one week prior. Subsequently the previously described finding of migrating nodular lesions were confirmed on one-month follow-up computed tomography (CT) scan.

The serology was eventually positive for schistosomiasis (IgE 556) and treatment was started (Praziquantel) with an interval of two weeks in between doses. On this therapy the patient improved with significant clinical response and a regression of CRP and eosinophilia.

## Discussion

Schistosomiasis is a parasitic infection caused by parasitic blood flukes acquired during contact with infested water and is known to be an important cause of disease in sub-Saharan Afrika. It is rarely seen in Europe except for occasional tourism associated exposures.

Pulmonary manifestations are most often seen after chronic hepatosplenic involvement with portal hypertension and development of collateral circulation. The eggs of the larvae are subsequently migrating to the alveolar spaces causing an eosinophilic inflammatory response. In this case the predominant HRCT finding of pulmonary involvement was the observation of diffuse nodular opacities which have already been described by Nguyen et al. in 2006 [1]. Our case places an emphasis on the migrating aspect of the nodular lesions (fading and growing) without implementing any medical therapy. Pulmonary tuberculosis, Pulmonary hemorrhage and cryptogenic organizing pneumonia were considered in the differential diagnosis of migrating lesions.

An interesting finding in this case is that the patient was recently tested positive for SARS-CoV-2 after having a high-risk contact (sub clinically). It is not clear if COVID-19 infection could trigger a host’s immune response to chronic indolent schistosoma infections or increase the morbidity and mortality of patients who are chronically exposed to the schistosoma parasite.
